# Food perception alone stimulates oviposition through serotonin signaling in *Drosophila*

**DOI:** 10.64898/2026.10.08.757633

**Published:** 2026-10-15

**Authors:** Tze Chang Ng, Madison Oh, Ayush Shrivastava, Christian Arntsen, Sai Sankpal, Matthew W. Chong, Advaitha Kamalakkannan, Jinhu Chung, Molly Stanley, Yang Lyu

## Abstract

Reproduction is strongly shaped by nutrient availability, but whether sensory information about food can regulate reproductive output before nutrients are consumed remains unclear. Here, using *Drosophila melanogaster* , we show that sensory perception of dietary yeast, a protein-rich food source widely used to stimulate fecundity in the fruit fly, is sufficient to rapidly promote egg laying. Specifically, we found that yeast odor exposure increased the rate of oviposition and the number of eggs laid within hours, independent of yeast intake, and that this response was mediated by olfactory signaling through the odorant receptor co-receptor Orco. We further identified serotonin receptor 2A (5-HT2A), but not 5-HT2B, as necessary for yeast odor-induced egg laying. We then asked whether yeast odor also elicits broader life-history changes and whether its reproductive and longevity effects depend on a shared mechanism. Consistent with previous observations, yeast odor also shortened lifespan. However, this lifespan effect was independent of 5-HT2A signaling, indicating that the reproductive and longevity responses to food perception are separable. Finally, our brain connectomic analyses revealed that yeast-responsive olfactory neurons preferentially influence oviposition descending neurons (OviDNs) compared with other olfactory neurons. OviDNs further connect with endocrine-related circuitry in the corpora allata (CA), and, most interestingly, the interneurons linking OviDNs to CA neurons are enriched for serotonergic and dopaminergic modulation. Taken together, our observations indicate that nutrient acquisition is not a prerequisite for animals to adjust their reproduction to the nutritional environment. Sensory information predicting food availability itself can elicit changes in neural state, likely through olfactory-reproductive circuits, that acutely alter reproductive physiology.

**Significance statement:**

Food perception can drive changes beyond feeding-related behaviors. This study establishes that olfactory perception of dietary yeast per se elicits egg-laying behavior in *Drosophila melanogaster* and that the conserved neuromodulator serotonin is required for this response. Our observations suggest that the reproductive state of animals is determined not only by commonly recognized factors such as ingested nutrients, but also by sensory cues that anticipate food availability.

